# In Silico Study on the Interactions, Molecular Docking, Dynamics and Simulation of Potential Compounds from *Withania somnifera* (L.) Dunal Root against Cancer by Targeting KAT6A

**DOI:** 10.3390/molecules28031117

**Published:** 2023-01-22

**Authors:** Sanjay H. Deshpande, Abdullatif Bin Muhsinah, Zabin K. Bagewadi, Gireesh M. Ankad, Mater H. Mahnashi, Deepak A. Yaraguppi, Ibrahim Ahmed Shaikh, Aejaz Abdullatif Khan, Harsha V. Hegde, Subarna Roy

**Affiliations:** 1 Department of Biotechnology, KLE Technological University, Hubballi 580031, Karnataka, India; 2 Department of Pharmacognosy, College of Pharmacy, King Khalid University, Abha 61441, Saudi Arabia; 3 ICMR-National Institute of Traditional Medicine, Belagavi 590010, Karnataka, India; 4 Department of Pharmaceutical Chemistry, College of Pharmacy, Najran University, Najran 66462, Saudi Arabia; 5 Department of Pharmacology, College of Pharmacy, Najran University, Najran 66462, Saudi Arabia; 6 Department of General Science, Ibn Sina National College for Medical Studies, Jeddah 21418, Saudi Arabia

**Keywords:** *Withania somnifera*, cancer, KAT6A, molecular docking, molecular dynamics

## Abstract

Cancer is characterized by the abnormal development of cells that divide in an uncontrolled manner and further take over the body and destroy the normal cells of the body. Although several therapies are practiced, the demand and need for new therapeutic agents are ever-increasing because of issues with the safety, efficacy and efficiency of old drugs. Several plant-based therapeutics are being used for treatment, either as conjugates with existing drugs or as standalone formulations. *Withania somnifera* (L.) Dunal is a highly studied medicinal plant which is known to possess immunomodulatory activity as well as anticancer properties. The pivotal role of KAT6A in major cellular pathways and its oncogenic nature make it an important target in cancer treatment. Based on the literature and curated datasets, twenty-six compounds from the root of *W*. *somnifera* and a standard inhibitor were docked with the target KAT6A using Autodock vina. The compounds and the inhibitor complexes were subjected to molecular dynamics simulation (50 ns) using Desmond to understand the stability and interactions. The top compounds (based on the docking score of less than −8.5 kcal/mol) were evaluated in comparison to the inhibitor. Based on interactions at ARG655, LEU686, GLN760, ARG660, LEU689 and LYS763 amino acids with the inhibitor WM-8014, the compounds from *W*. *somnifera* were evaluated. Withanolide D, Withasomniferol C, Withanolide E, 27-Hydroxywithanone, Withanolide G, Withasomniferol B and Sitoindoside IX showed high stability with the residues of interest. The cell viability of human breast cancer MCF-7 cells was evaluated by treating them with *W. Somnifera* root extract using an MTT assay, which showed inhibitory activity with an IC50 value of 45 µg/mL. The data from the study support the traditional practice of *W*. *somnifera* as an anticancer herb.

## 1. Introduction

Cancer is defined by the excessive proliferation of abnormal cells with elevated levels of replication and invasion ability. In the year 2020, a total of 19.3 million cases were diagnosed along with 10 million deaths, based on present data. It is predicted that the cancer burden will increase to 28.4 million cases by the year 2040, a jump of 47% from the year 2020 [1]. Despite extensive attempts and the availability of multiple chemotherapeutic agents, their efficacies are still limited because of heterogeneity in cancer cells, emergences of drug-resistant populations of cells and complicated tumor interactions [2]. Natural compounds with medicinal properties have drawn interest as chemotherapeutics due to their ability to target various cancer growth mechanisms, including cell proliferation and resistance to death, replicative immortality, invasion and metastasis, angiogenesis and inflammation that promotes tumors [3]. Several anticancer targets have been studied, but in recent times, the selection of targets based on the role of proteins in the initiation and development of cancer has been considered critical, which makes epigenetic proteins very important therapeutic targets. One such important group of epigenetic factors are the lysine acetyltransferase (KAT) enzymes which are involved in the mechanism of chromatin assembly regulation, DNA repair, RNA transcription and reactions managed by DNA via lysine side-chain acetylation of transcription factors and histones [4]. The connection between irregular acetylation of proteins and the genesis of many diseases has prompted research on KAT inhibitors (KATi) with the ultimate aim of finding therapeutics. Considering the pivotal role of KATs, proteins such as KAT6A—which is involved in major cellular pathways and in the regulation of transcriptional factors and has an oncogenic nature—are important targets in cancer treatment. Inhibiting the activity of KAT6A provides a way to stop the cell cycle which also induces cell cycle exit and cellular senescence. The KAT inhibition strategy to treat cancer is in its initial stages with very few candidates such as WM-8014 for KAT6A and KAT6B inhibition [5]. KAT6A consists of PHD finger, MYST family zinc finger and MOZ/SAS family domains. The domains that are ligandable/druggable are the MYST family zinc finger and MOZ/SAS family domains [6]. According to The Human Protein Atlas, KAT6A is characterized as a cancer-related gene, metabolic protein, transcription factor, proto-oncogene and potential drug target. KAT6A is localized to nucleoli and cytosol [7]. Medicines derived from plant sources majorly contributed to primary healthcare during ancient times and most of them have been documented based on empirical knowledge [8]. The knowledge to use plants therapeutically has led to the discovery of many modern drugs, which are now being scaled up using tissue culture technology and also by modern agricultural techniques. Plants—being highly variable and sensitive in nature, the production of therapeutically efficient bio actives is very difficult. Many new technologies have been employed to obtain a consistent production of bioactive compounds [9]. An increase in demand and production has had a great impact on related research, with fast-paced extensive research being conducted to discover new potential drugs and to evaluate the activity and pharmacological nature of phytocompounds [10]. Several plants have been prioritized as important medicinal plants in terms of their application and also based on their ease of growing with a potential market. Plants such as *Acacia catechu* (L. f.) Willd, *Aegle marmelos* (L.) Correa, *Andrographis paniculata* (Burm. f.) Wall, *Berberis aristata* DC, *Garcinia indica* Choisy, *Gymnema sylvestre* (Retz.) R. Br, *Ocimum sanctum* L., *Phyllanthus emblica* L., *Sarac aasoca* (Roxb.) De Wilde and *Withania somnifera* (L.) Dunal have been identified as prioritized species for medicinal plant research, out of which *Saraca asoca* and *Withania somnifera* are known to have anticancer activity based on ethnomedicinal practices [11]. *S. asoca* is one of the most threatened medicinal plants with a high level of adulteration in the market due to its demand. The source of *S. asoca* is majorly within protected forests, and *S. asoca* has been listed as a vulnerable species by the IUCN (https://www.iucnredlist.org/fr/species/34623/9879360, accessed on 1 January 2022). The concentration and production of phytocompounds in *S. asoca* vary based on their genotypes and growth conditions, making it a very intricate plant for research. In comparison to *S. asoca*, *W. somnifera* is not a forest species, and has been cultivated as a commercial medicinal plant for a long time. Hence, in this study, *W. somnifera* was considered for evaluation of anticancer activity, which can be commercially and quantitatively feasible in medical applications. *Withania somnifera* (L.) Dunal (Solanaceae), also known as “Ashwagandha” is an important medicinal plant that has been widely researched and documented. *W. somnifera* is a plant that typically originated from regions of Asia and Africa with dry climatic conditions (India, Afghanistan, South Africa and Middle Eastern countries) [12] with a wide spectrum of therapeutic applications [13,14,15]. The first report on the medicinal properties of *W. somnifera* in a publication was by Devi et al. [16], in which alcoholic extract from the roots of *W. somnifera* was intraperitoneally injected into Balb/c mice, which led to the regression of sarcoma 180 cells. Since then, a number of research projects have shown that *W. somnifera* has immense potential as an effective anticancer agent [17]. A wide range of therapeutic uses from ethnic practices is documented in the Database of Ethno-Medicinal Plants of Western Ghats (https://nitmmedplantsdb.in, accessed on 18 January 2022). Some studies with *W. somnifera* extracts have shown potent anticancer efficacy against several cancers such as breast, cervical, pancreatic, lung, skin, etc. [18]. Taxonomical evidence states that *W. somnifera* is known to have a rich source of compound classes called withanolides. These classes of compounds are known to show diverse pharmacological effects, which include anti-inflammatory, hepatoprotective, antitumor, antimicrobial, anti-diabetic and immunosuppressive activities [19,20,21]. Studies have shown that withanolides from the roots of *W. somnifera* are known to have an inhibitory action on the transcription signal and signal transducer in human breast cells [22]. Extracts of *W. somnifera* have been shown to have significant anti-proliferative potential against breast cancer cells, even in small doses, wherein the IC50 value of extracts was found to be 9.014 µg/mL [23]. Some studies have been published on the associated anticancer activities but the exact mechanism of the bioactive compounds in complex with cancer targets is sparsely studied. Hence, to elucidate the interactions and the stability of compounds in complex with the target protein, an herbal informatics approach can be employed. An herbal informatics approach includes the identification of a therapeutically important anticancer target, molecular docking studies with the bioactive compounds from the herbs, ADMET studies of the compounds and molecular dynamics studies [24]. The structure–activity relationship and anticancer potential of W. somnifera is well-documented in the literature. The withanolides and withaferin A possess binding energies against several proteins, which can form potential leads for cancer therapy [25]. A comprehensive study of the phytocompounds from the roots of *W. somnifera*, in comparison with existing inhibitors, can help in the assessment of *W. somnifera* as a potential anticancer herb by elucidating the possible interactions with the KAT6A protein via in silico studies.

## 2. Results

### 2.1. ADMET Properties

**Figure 1:**
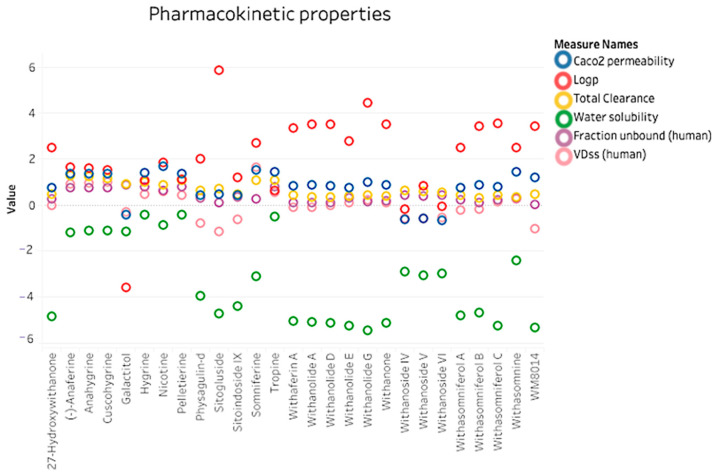
Pharmacokinetic properties plot showing the comparision between the compounds from *W. somnifera* and inhibitor (WM-8014) using pkCSM server.

The canonical SMILES of selected phytocompounds and the inhibitor (WM-8014) were submitted to the pkCSM server. The resulting data were tabulated and plotted according to their properties. The data analysis of results is plotted as shown in Figure 1 and complete details of the properties are provided in Supplementary Data.

It was observed that the compounds from *W. somnifera* have better pharmacokinetic properties in comparison to the inhibitor WM-8014. It was observed that the compounds from *W. somnifera* have higher water solubility, permeability and better clearance.

### 2.2. Binding Site Assignment

**Figure 2:**
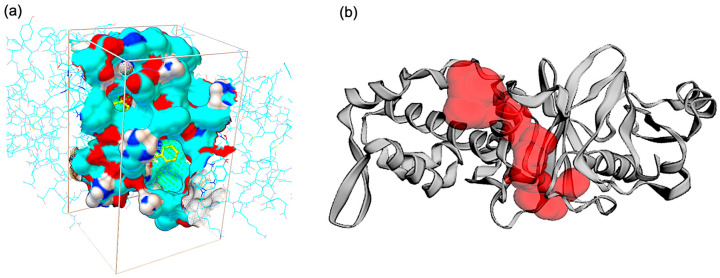
(**a**) Binding cavity and grid box of Lysine acetyltransferase 6A domain, defined based on the co-crystalized ligand and importance residues in catalytic activity; (**b**) Solvent-accessible surface area of the defined cavity.

The structure of KAT6A, co-crystallized with the ligand WM-8014 available on PDB, was considered for the binding site. Based on the interaction plot of KAT6A and WM-8014 (PDB ID: 6OIO), interaction sites with hydrophobic interaction (LEU601, ILE649, ARG660, LEU680), hydrogen bonds (ARG655, GLY657, GLY659, ARG660, SER690) and water bridge interaction (ARG656) were identified (Figure 2). The binding site of acetyl-CoA is the most critical part of lysine acetyltransferase (KAT6A) activity [26]. Considering all the important residues obtained from the co-crystallized ligand and the literature, a docking grid box was created. The grid was generated with a set of coordinates (x = 3.82, y = 11.14, z = 18.848) using the Autosite tools program; the created grid is shown in Figure 2a. The solvent-accessible surface area of the protein was calculated using the CastP analysis server. The analysis showed that the protein has a total solvent-accessible area of 703.852 Å^2^ and a volume of 414.188 Å^3^ for the single pocket predicted. The solvent-accessible pocket is depicted in Figure 2b.

### 2.3. Molecular Docking Results

In search of interactions between the active site and important residues of KAT6A (lysine acetyltransferase 6A domain) and the compounds from *W. somnifera*, molecular docking studies were carried out for 27 phytocompounds and the validated inhibitor. The ranking of protein–ligand complexes was conducted based on the docking score (binding energy). Compounds with a docking score of less than −8.5 kcal/mol were considered for further evaluation and a 3D representation of the complexes is given in Supplementary Material Figure S1. A comprehensive evaluation of all the docked compounds was conducted based on the binding energy and the interaction of key residues within the binding cavity in comparison to co-crystallized ligand interactions (Table 1). Two-dimensional structures of the compounds from *W. somnifera* are given in Supplementary Figure S5. The binding energy data for all the compounds are provided as Supplementary Table S1.

#### Molecular Dynamics (MD)

**Figure 3:**
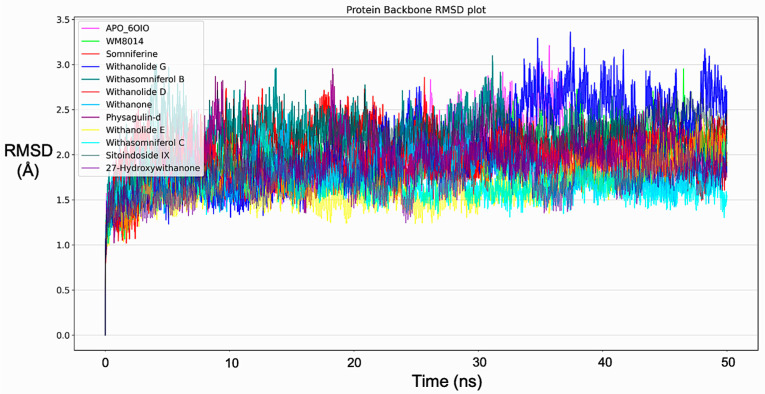
RMSD plots of protein backbone from Molecular dynamics for all protein–ligand complexes, including inhibitor (WM-8014).

**Figure 4:**
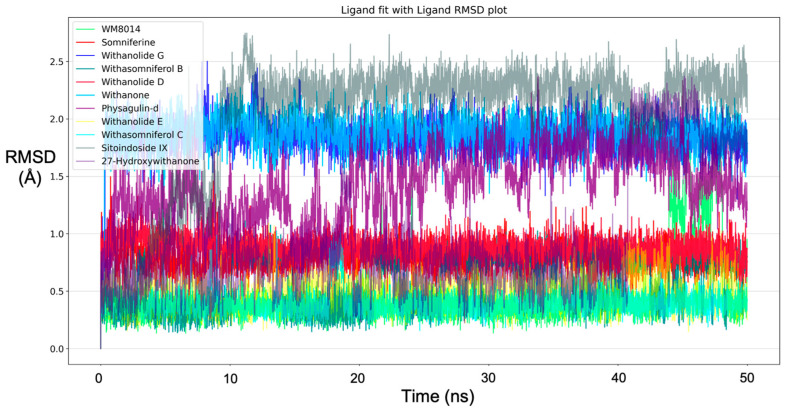
RMSD plot for ligands from Molecular dynamics for all phytocompounds and the inhibitor (WM-8014).

Molecular dynamics simulation for all top-ranking complexes based on the binding energy and the important interactions was carried out with Desmond for a duration of 50 ns (5000 frames). The data from the trajectory were analyzed and tabulated as shown in Table 2. The RMSD values of the protein backbone for all complexes were plotted and are shown in Figure 3 and the RMSD plot for ligands complexed with the protein is shown in Figure 4. The ΔG (total binding energy) of the system from the MMGBSA analysis was tabulated (Table 2). The ligand structures from the beginning of the simulation and after the simulation were superimposed and structures have been given in Supplementary Figure S4; RMSF values are given in Supplementary Table S2. Based on the RMSF values observed, it was found that compounds from *W. somnifera* remained highly stable and in sync with the inhibitor WM-8014.

After the simulation, the total binding energy (ΔG) was calculated from MMGBSA analysis for all complexes. MMGBSA analysis showed that ΔG of the inhibitor WM-8014 was higher, followed by Withanolide D, Sitoindoside IX and Somniferine, as shown in Table 2. The RMSD values of the protein backbone were plotted (Figure 3), in which the mean RMSD value of the protein backbone in complex with phytocompounds showed a lower mean in comparison to the inhibitor and the individual compound; plots have been given in Supplementary Figure S2. Withanolide E, Withasomniferol C, 27-Hydroxywithanone, Withanone, Withanolide D and Physagulin-d showed lower mean RMSD than the inhibitor for the protein backbone. The RMSD plot of ligands in complex with proteins was plotted and Withasomniferol C showed a lower RMSD mean than the inhibitor, showing its stability during the simulation (Figure 4). Individual plots have been given in Supplementary Figure S3.

**Figure 5:**
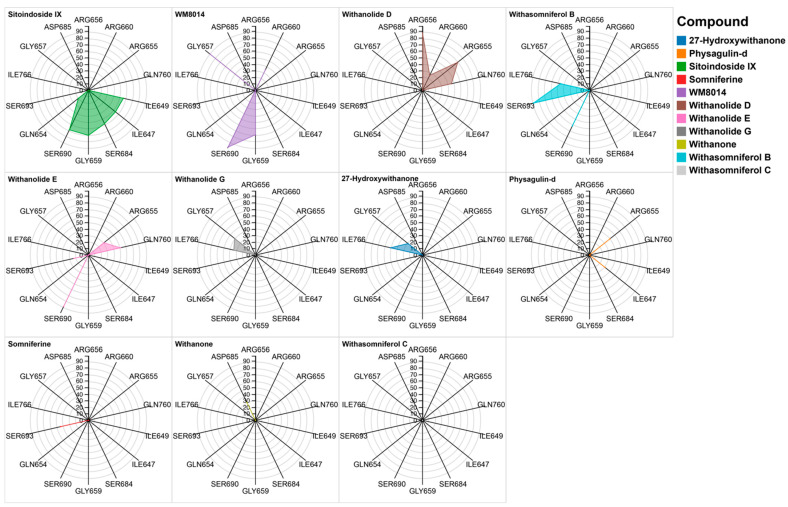
Radial plot for all the compounds, including the inhibitor WM-8014, showing the percentages of contacts during simulation.

Ligand contact points during the simulation were tabulated and the percentages of the duration of the contacts with KAT6A were analyzed. The contact map analysis of every compound, including the inhibitor WM-8014, was plotted as a radial distribution plot (Figure 5).

**Figure 6:**
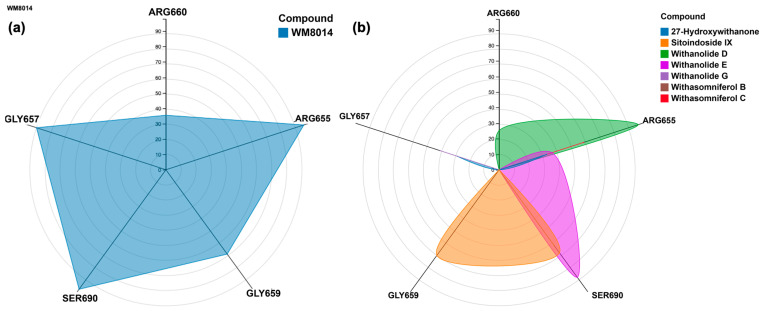
(**a**) Radial distribution plot for the percentages of contact during the simulation for WM-8014; (**b**) Radial distribution plot for the percentages of contact during the simulation for the compounds from *W. somnifera* with the same contact points as WM-8014.

**Figure 7:**
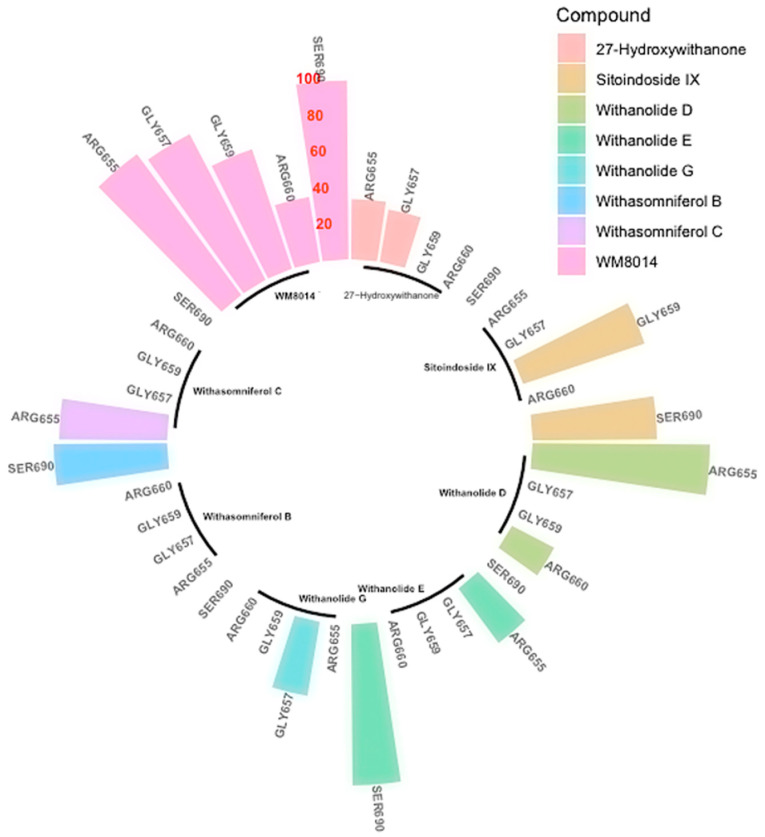
Contact points and percentages of contact duration for the compounds from *W. somnifera* and inhibitor WM-8014 (Reference percentages have been marked in red colour entries over one of the spoke).

**Figure 8:**
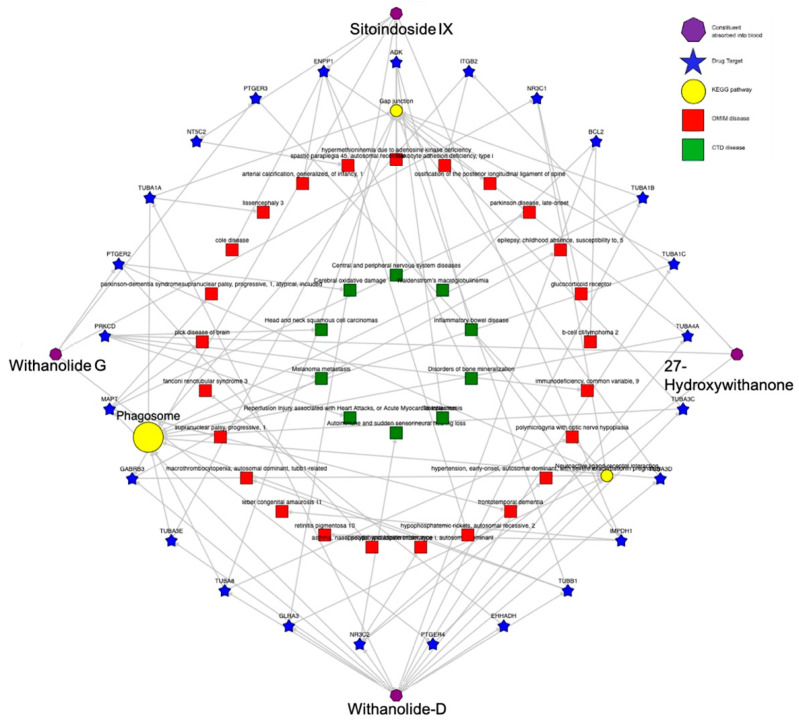
Network of compound–target–pathway–disease for the top compounds from *W. somnifera*.

Further, ligand contact points during the simulation were analyzed specifically for amino acid residues ARG655, LEU,686, GLN760, ARG660, LEU689 and LYS763, which had contact points during the simulation of inhibitor WM-8014. Many compounds from *W. somnifera* showed contact points very similar to those of the inhibitor WM-8014. Withanolide D showed interactions at ARG656, ARG655, ARG660 and GLN760; Withanolide E showed interactions at ARG655, GLN760, SER690 and SER693; Withasomniferol C showed an interaction at ARG655; 27-Hydroxywithanone showed interactions at ARG655, ILE766 and GLY657; Withanolide G showed an interaction at GLY657; and Sitoindoside IX showed interactions at ILE649, ILE647, SER684, GLY659, SER690 and GLN654. Based on the protein and ligand mean RMSD, Withanolide E and Withasomniferol C had stable and lower RMSD values in comparison to the inhibitor WM-8014. The contact map analysis from the simulation trajectory of Withanolide E showed long contact durations at the sites ARG655, SER690, SER693 and GLN760; similarly, for Withasomniferol C, contacts were seen at ARG655 for almost the entire duration of the simulation. Radial distribution plots were separately generated for the inhibitor WM-8014 (Figure 6a) and the compounds from *W. somnifera* (Figure 6b). The contact points of ligands and the percentages of contact duration are depicted in Figure 7. Based on the top compounds with active site interactions, the compounds were subjected to network analysis and a network was constructed, as shown in Figure 8. The network was constructed based on the criteria in which a compound targets a minimum of 10 targets with 0.0.5 probability. From the network, it was observed that compounds were enriched against head and neck squamous cell carcinomas, inflammatory bowel disease, central and peripheral nervous system diseases, melanoma metastasis and hypertriglyceridemia. Even based on network pharmacology analysis, it was clearly seen that compounds from *W. somnifera* target different types of cancer and associated diseases.

To confirm the reliability of the docking studies and simulation studies, the crystallized PDB structure (PDB id: 6OIO) of WM-8014 in complex with KAT6A was subjected to molecular dynamics with the same conditions used for the docked complexes. The contact map analysis from the molecular dynamics of the crystallized structure of inhibitor WM-8014 and KAT6A showed contacts at ARG655, GLY659, ARG660 and SER690 for almost the entire duration of simulation, thereby confirming the correctness of the molecular dynamics simulation studies. The total energy of the systems was calculated and it was clearly seen that the energy values are very similar, showing stability and similarity (Table 3). To reconfirm the stability of the simulation, the top three compounds (Withanolide E, Withanolide D and Sitoindoside IX) with the greatest interaction with the active sites were further subjected to 100 ns of simulation. The simulation clearly showed high level of stability (Supplementary Figure S6).

**Figure 9:**
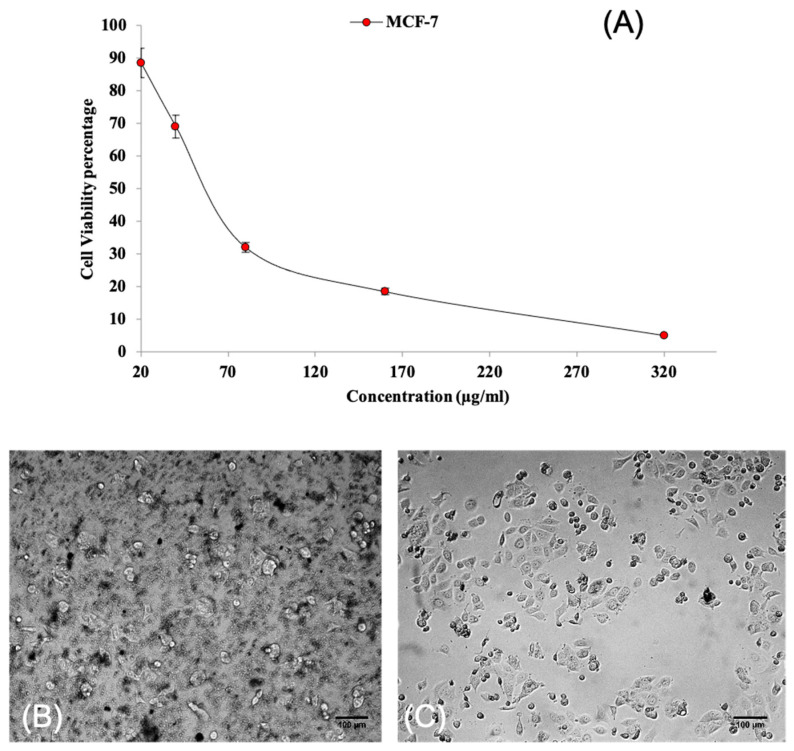
Anticancer activity of root extract of *W. Somnifera* against human breast cancer MCF-7 cell line. (**A**) Cell viability percentage; (**B**) extract-treated cancer cells; (**C**) Cisplatin-treated cancer cells.

The cell viability of human breast cancer MCF-7 cells was evaluated after treatment with root extract of *W. Somnifera* via MTT assay. The anticancer effect of the roots of *W. Somnifera* was clearly evidenced on human breast cancer MCF-7 cell lines under the studied concentration range: 20–320 µg/mL. The dose-dependent decline in the cell viability percentage is shown in Figure 9A. The drastic decline in cell viability is evidenced beyond an effective concentration of 80 µg/mL. The IC50 value, which represents 50% inhibition in cell growth, was found to be 45 µg/mL. The inhibitions in cell growth caused by the effect of root extract of *W. Somnifera* are also depicted in Figure 9B. Microscopic observation reveals the rupturing of cancer cells with effective treatment of root extract of W. Somnifera. However, the untreated cells remained undisturbed. Figure 9C depicts treatment with cisplatin (standard), which shows a similar effect as that of the extract. The results strongly indicate that the roots of *W. Somnifera* possess strong anticancer compounds such as different withanolides. The anticancer effect of the roots can be attributed to the presence of bioactive compounds, namely Withanolide D, Withasomniferol C, Withanolide E, 27-Hydroxywithanone, Withanolide G, Withasomniferol B and Sitoindoside IX. The outcomes of in silico results are validated with supplementary in vitro studies.

## 3. Discussion

Considering all the domains of KAT6A, WM-8014 in its crystallized form takes up the position of the acetyl-CoA-binding site present on KAT6A. The site is partially enclosed between the α-helix of the main protein by residues ASP685 to ARG704 and the loop which extends from GLU654 to GLY657 [5]. Similarly, compounds from *W. somnifera* also have interactions at similar points during the simulation, thereby predicting their potent inhibitory action on KAT6A, which might be synergistic in nature or by individual action. Bioactive compounds from *W. somnifera* are known to have inhibitory effects on breast cancer cell lines. Several inhibitory actions have been postulated including PAR-4 induction, formation of ROS (reactive oxygen species), proteasome inhibition and inhibition of heat shock protein 90 (HSP90). HSP90 inhibition by withanolides in cancer cells induces a cytotoxic effect on the cancer cells, thereby reducing cancer [27]. Some animal studies on acute myeloid leukemia have been carried out to identify inhibitors of KAT6A that induce senescence and arrest growth in lymphoma [28]. Even a partial blockage of KAT6A can help reduce the proliferation of MYC-induced lymphoma and acute myeloid leukemia [29]. In one of the studies, root extracts from W. somnifera were reported to have inhibitory activity for lipogenesis in 22Rv1 cells, possibly by downregulating c-Myc and p-Akt levels, suggesting that fatty acid metabolism may have a role in cancer cells and antitumor activity in prostate cancer [23]. Traditionally, *W. somnifera* has been used widely in many therapeutic applications. One such application of *W. somnifera* is its usage as a rejuvenator which helps in increasing energy levels and mitochondrial health [30], which can be beneficial in anticancer activity. Another application lies in the fact that it has been traditionally used a immunomodulator [31]. The study provides insights into the compounds of *W. Somnifera* roots as potential therapeutic molecules harboring anticancer attributes for the target KAT6A. The study also validates the ethnomedicinal use of *W. somnifera* as a potential anticancer herb. A similar study was conducted by Gurav et al. (2023) [32] to investigate the ethnopharmacological properties of *W. somnifera* for its aphrodisiac potential by employing in silico, in vitro and in vivo strategies, and the results of molecular modeling were in agreement with the biological activity. Extracts from W. somnifera roots have also shown cytotoxic activity on lung adenocarcinoma cells with an IC50 of 99.7 μg/mL [33]. A subcritical water extraction method has been reported by Nile et al. (2019) [34] that showed good concentrations of withanosides, withanolides and steroidal lactone compounds with promising biological activities. Considering the results and based on the studies conducted previously, *W. somnifera* can be considered for anticancer therapeutic applications.

## 4. Materials and Methods

### 4.1. Data Source

In the current study, the list of known phytocompounds from the roots of *W. somnifera* were listed out from PubChem [35], ChEBI [36] and IMPATT databases [37]. The non-duplicate list of phytocompounds was taken as input to download the structural file of each compound. The structural file of each compound was downloaded from the PubChem database iteratively using a Python script available on GitHub (https://github.com/sandes89/PubDown, accessed on 10 February 2022). A total of twenty-six compounds were downloaded from the database and the complete list with their physicochemical properties is given in the Supplementary Data (Table S1).

### 4.2. Prediction of ADMET Properties and Network Analysis

The canonical SMILES of the compounds were tabulated from the PubChem database [35] and the list of canonical SMILES was submitted to pkCSM [38]. The portal assesses the structure of the compounds and provides pharmacokinetics and pharmacodynamics data for each compound. The analysis provides information on colon permeability (Caco-2), intestinal absorption, water solubility, BBB permeability, cytochrome P450 inhibition, CYP substrates, total clearance and toxicity. The compounds were subjected to network analysis to understand the possible action of compounds against the targets and the corresponding pathways and diseases. The network analysis was carried out using data from the BindingDb database [39] and the network was constructed using Cytoscape version 3.6.1 [40]. 

### 4.3. Molecular Docking

#### 4.3.1. Protein Preparation

**Figure 10:**
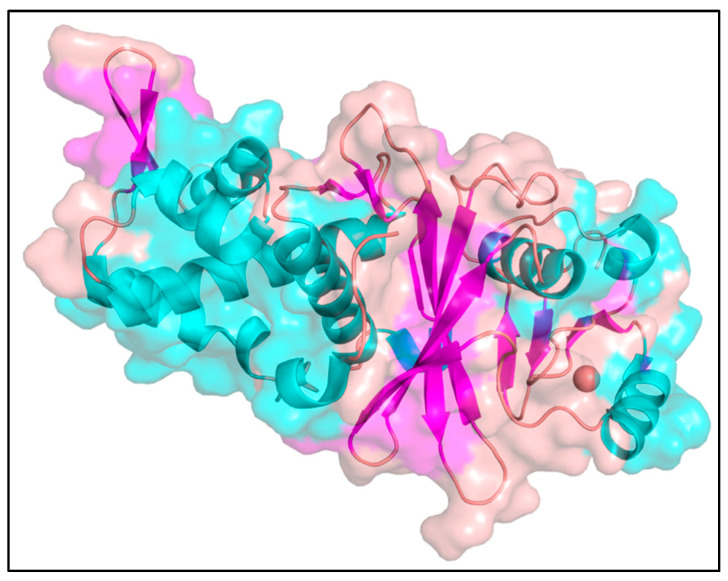
MYST acetyltransferase domain (KAT6A) (PDB id: 6OIO) as a target protein for docking studies.

The crystal structure of MYST acetyltransferase domain 6A (KAT6A) (PDB id: 6OIO) (resolution: 1.70 Å) [41] was downloaded from RCSB PDB (Protein Data Bank) [42] Figure 10. A graphical user interface of AutoDock tools was used to prepare the protein in PDBQT format. The co-crystallized water molecules and other atoms were removed, apart from the protein residues and Zn ion in the structure. The clean protein molecule was added with Kollman charges, parameters related to solvation, and polar hydrogens. Once all the parameters were added, the protein was considered a rigid molecule and saved in PDBQT format for molecular docking studies. The protein minimization was carried out using Desmond by running a minimization run of 100 ps before taking it for molecular docking analysis.

#### 4.3.2. Identification of Binding Cavity

The most crucial aspect of molecular docking studies is the identification of active residues and a cavity for a ligand to bind. In this study, the PDB structure selected was MYST acetyltransferase domain 6A (KAT6A) (PDB id: 6OIO) having co-crystallized ligand WM-8014 (PDB id: ML7). The co-crystallized protein and the ligand were analyzed using Autosite [43]; based on the existing information of ligand-binding sites, the active cavity points were identified. Autosite mainly employs feature point-based prediction to identify the binding cavity of protein. Furthermore, domain-based identification of the active residues was employed by considering the data available on Uniprot [44] and Prosite [45]. The solvent-accessible area of the protein was calculated using the CastP server [46]. The secondary structure components of KAT6A protein structure were annotated, which is shown in Figure 10.

#### 4.3.3. Ligand Preparation

The POAP tool [47] was used to prepare the compounds for geometrical optimization. A total 5000 steps of minimization were conducted using the steepest descent method, a MMFF94 force field was employed and hydrogens were added. A total of 50 conformers for each ligand were generated, out of which the best was considered for the molecular docking studies [48]. The compound with the lowest energy and valid geometry after minimization was considered for the molecular docking studies. The conformer of the inhibitor was directly used from the co-crystallized structure, as it directly represents the biological form of the compound.

#### 4.3.4. Docking of the Compounds Using the POAP Program

The compounds with optimized geometry and shape were subjected to molecular docking with the lysine acetyltransferase domain. Ligands were considered as flexible, and the protein as a rigid molecule. The grid box was prepared in a Dfrgui [49] based on the active residues identified from Autosite and the literature. The docking was carried out at an exhaustiveness of 100 in vina [50]. The resulting protein–ligand complexes were analyzed using PLIP (Protein–Ligand Interaction Profiler) [51].

### 4.4. Molecular Dynamics (MD)

MD simulations for the protein–ligand complexes were conducted using Desmond’s explicit solvent MD package (Desmond Molecular Dynamics System, D. E. Shaw Research, 2016) with a built-in, optimized fluid simulation potential (OPLS 2005) force field [52]. An additional protein was subjected to restrained minimization for hydrogen atoms by converging heavy atoms to a RMSD (root mean square deviation) value of 0.30 Å [53,54]. The system was set up for simulation using a predefined SPC water model (simple point charge) in a cubic box with periodic boundary conditions and with a size of 10 Å × 10 Å × 10 Å. Neutralization of the system was carried out with 0.15 M NaCl (physiological concentration of monovalent ions). System relaxation was accomplished through the hybrid implementation of steepest descent and Broyden–Fletcher–Goldfarb–Shanno limited-memory algorithms [55]. Molecular dynamics for the system generated was carried out for 50 ns and the conditions for the system were set at a temperature of 300 K using the thermostat model and a pressure of 1.01325 bar using the isothermal–isobaric ensemble class (NPT); trajectory was measured at every 100 ps recording interval, with 5000 frames of data. After simulation, MMGBSA calculation was carried out by employing a MMGBSA.py script to the output trajectory to calculate total binding energy (ΔG) [56]. The total energy of the system was calculated using the Desmond simulation quality analysis tool.

### 4.5. In Vitro Studies

#### 4.5.1. Materials Used

Authenticated root samples of *Withania somnifera* (L.) Dunal were collected from the nursery of the University of Agricultural Sciences, Dharwad, and identified based on taxonomy. The specimen voucher was deposited at the Department of Biotechnology, KLE Technological University, Hubballi, Karnataka, India. The standards and chemicals employed in the present study were procured from Sigma-Aldrich Pvt Ltd. (Burlington, MA, USA). Human breast cancer MCF-7 cell lines were used for in vitro anticancer studies.

#### 4.5.2. Extraction Method

The roots of *W. Somnifera* were washed repeatedly with sterile distilled water to remove the soil particles and allowed to shade dry at room temperature. The dried roots were powdered and sieved and employed for aqueous extraction using a Soxhlet extractor. The extraction was carried out at 70 °C for 5 h and concentrated using a rotary evaporator [57]. The extract was used for further analysis of anticancer activities.

#### 4.5.3. Analysis of Anticancer Properties by 3-(4, 5-Dimethylthiazol2-yl)−2,5-Diphenyltetrazolium Bromide (MTT) Assay

The in vitro assessment of the anticancer activities of W. Somnifera roots was carried out according to the method described by [57] using an MTT assay procedure. The root extract of *W. somnifera* was evaluated experimentally for cell viability of human breast cancer MCF-7 cell lines. Briefly, the MTT assay was carried out as follows: the human breast cancer MCF-7 cells were seeded for 24 h in microplates. The cells were further treated with different concentrations (20–320 µg/mL) of *W. Somnifera* root extract. The cells were washed with phosphate-buffered saline (PBS) and MTT was added in each well followed by incubation for 5 h. Dimethyl sulfoxide (DMSO) was added to solubilize the formazan crystals and absorbance was measured at 570 nm. The control represents the cells without the treatment with extract and possessed 100% cell viability. The percentage cell viability of extract-treated cells and the IC50 value were calculated. The assay was carried out in triplicates using cisplatin as the standard drug.

## 5. Conclusions

*W. somnifera* has earned attention from the scientific community for its cancer-preventive molecules. Several of this plant’s active compounds have been effectively isolated and screened and are known to have inhibitory or preventive activity against cancer cells. Although *W. somnifera* has been extensively used for its therapeutic properties in traditional medicines, very few studies have examined the mechanism of its specific action against cancer cells. The in silico study conducted here compares the interaction of KAT6A with the selective inhibitor and the bioactive compounds from the roots of *W. somnifera.* The study showed very encouraging results in terms of binding affinity and molecular dynamics of bioactive compounds, namely Withanolide D, Withasomniferol C, Withanolide E, 27-Hydroxywithanone, Withanolide G, Withasomniferol B and Sitoindoside IX, from the roots of *W. somnifera* in comparison to the validated inhibitor. From this study, we can determine that the bioactive compounds from *W. somnifera* can be potential therapeutic molecules for KAT6A, which is a HAT oncogene. The compounds from *W. somnifera* can also be used in combination with the inhibitor WM-8014 to increase the efficacy and potency of cancer treatment. The findings in this study not only validate the ethnomedicinal use of *W. somnifera* as an anticancer herb by predicting the possible modes of action by inhibiting the key residues of KAT6A, but can also encourage in vitro and in vivo study evaluations of the same kind with the identified phytocompounds. However, to extrapolate the results of the present study to clinical trials and drug development, further experimental studies are warranted. The results obtained from the present study support the traditional use of roots of *W. somnifera* as an anticancer herb, which is supported by in-vitro experiments. 

## Figures and Tables

**Table 1:** Binding energy values and Binding site interactions from molecular docking of *W. somnifera* root compounds with KAT6A.

Compound	Binding Energy (Kcal/mol)	Interactions		
		Hydrophobic Interaction	Hydrogen Bond	Salt Bridge
WM-8014	−10.8	LEU601, ILE649, ARG660	ARG660, GLY657, GLY659, ARG690	
Somniferine	−9.6	GLN760, LYS763	ARG655, ARG656, GLY657, TYR658, GLY659	
Sitoindoside IX	−9.6	ARG660, LYS763	ILE649, GLN654, SER684, LYS763	
Withasomniferol B	−9.2	ARG655, ARG660, LEU689, TRP697	ARG655, ARG660	
Withanolide G	−9.1	ARG660, LEU689, TRP697, LYS763	ARG655, GLY657	
Withanone	−9	ARG660, LEU689, TRP697, LYS763	ARG655, GLY657, LYS763	
Withanolide E	−8.8	ARG660, LEU689, LYS763, PRO765	ARG655, GLY657, GLN760	LYS763
Physagulin-d	−8.6	ARG660, ILE663, LEU686, LYS763	ILE647, ILE649, GLN654, SER684	
Withasomniferol C	−8.6	ARG660, LEU689, LYS763	ARG655, GLY657, ARG660	ARG655
Withanolide D	−8.5	ARG660, GLN760, LYS763	ARG655, ARG656, GLY657, TYR658, GLY659	
27-Hydroxywithanone	−8.5	ARG660, LEU689, ARG692, TRP697, GLN760, LYS763	ARG655, GLY657, GLN760	

**Table 2:** MD simulation and analyzed parameters.

Compound	Total Atoms	Protein RMSD (Å) (Mean)	Ligand RMSD	ΔG (Total Binding Energy) Mean
			(Å) (Mean)	
WM-8014	37,402	1.96	0.43	−88.38
Somniferine	34,529	2.06	0.79	−59.47
Withanolide G	34,409	2.06	1.89	−41.29
Withasomniferol B	37,273	2.16	0.56	−47.62
Withanolide D	34,544	1.90	0.88	−71.16
Withanone	34,409	1.76	1.87	−35.76
Physagulin-d	34,457	1.94	1.42	−41.30
Withanolide E	34,416	1.65	0.49	−48.91
Withasomniferol C	34,418	1.69	0.39	−41.77
Sitoindoside IX	34,448	2.14	2.00	−60.39
27-Hydroxywithanone	34,542	1.77	0.93	−52.60

Protein RMSD—Protein (Root mean square deviation); Ligand RMSD—Ligand (Root mean square deviation); ΔG—Binding free energy from MMGBSA analysis.

**Table 3:** Total energy of the systems subjected to molecular dynamics.

Compounds	Total Energy (kcal/mol)	Std. Deviation
Withanolide D	−89,776.661	122.76
Sitoindoside IX	−89,993.379	121.87
Withanolide E	−89,973.926	123.642
Physagulin-d	−90,020.423	122.803
Somniferine	−89,748.161	120.236
27-Hydroxywithanone	−89,940.324	122.504
Withanolide G	−89,971.235	123.043
Withanone	−90,033.506	121.551
Withasomniferol B	−89,663.812	125.308
Withasomniferol C	−97,730.765	127.169
WM8014	−97,754.831	125.992
APO_60IO	−90,298.600	122.209

## Data Availability

Data will be made available if required.

## Supplementary Materials

The following supporting information can be downloaded at: https://www.mdpi.com/article/10.3390/molecules28031117/s1: Figure S1: 3D representation of docking complexes of MYST Histone acetyltransferase domain with ligands (a) WM8014, (b) Somniferine, (c) Sitoindoside IX, (d) Withasomniferol B, (e) Withanolide G, (f) Withanone, (g) Withanolide E, (h) Physagulin-d, (i) Withasomniferol C, (j) Withanolide D, (k) 27-Hydroxywithanone; Figure S2: Individual RMSD plots of protein backbone from Molecular dynamics for all protein–ligand complexes; Figure S3: Individual RMSD plot for ligands from Molecular dynamics for all phytocompounds and the inhibitor; Figure S4: Superimposed structures of compounds (First and last frame of molecular dynamics) from Withania somnifera, which showed promising results with RMSF values. Figure S5: 2D structures of compounds from Withania somnifera subjected to molecular dynamics; Figure S6: RMSD plots of Withanolide D, Withanolide E and Sitoindoside IX in complex with KAT6A systems for duration of 100 ns; Table S1: Predicted physicochemical properties of phytocompounds and standard drug; Table S2: RMSF values of compounds before and after simulation having the best results.
