# Supplementary material for: In Silico Study on the Interactions, Molecular Docking, Dynamics and Simulation of Potential Compounds from Withania somnifera (L.) Dunal Root against Cancer by Targeting KAT6A
Source: Molecules. 2023 Jan 22;28(3):1117. doi: 10.3390/molecules28031117 (PMC9920226; doi:10.3390/molecules28031117)
Supplement: Supplementary file 1 [file molecules-28-01117-s001.zip › molecules-2107740-supplementary.pdf]

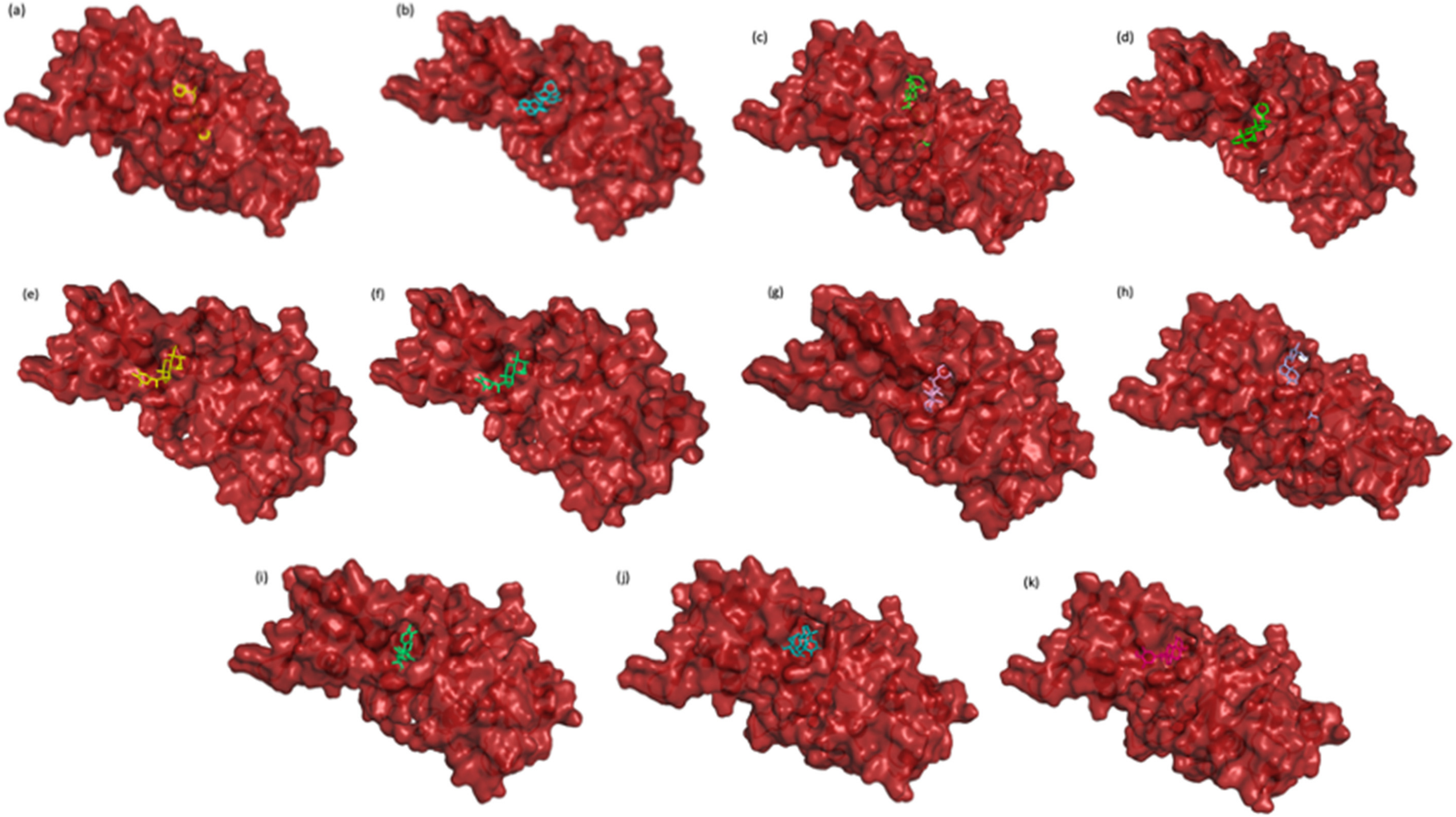

Supplementary Figure S1: 3D representation of docking complexes of MYST Histone acetyltransferase domain with ligands (a) WM8014 (b)Somniferine (c) Sitoindoside IX (d) Withasomniferol B (e) Withanolide G(f) Withanone (g) Withanolide E (h) Physagulin-d(i) Withasomniferol C (j)Withanolide D (k) 27-Hydroxywithanone

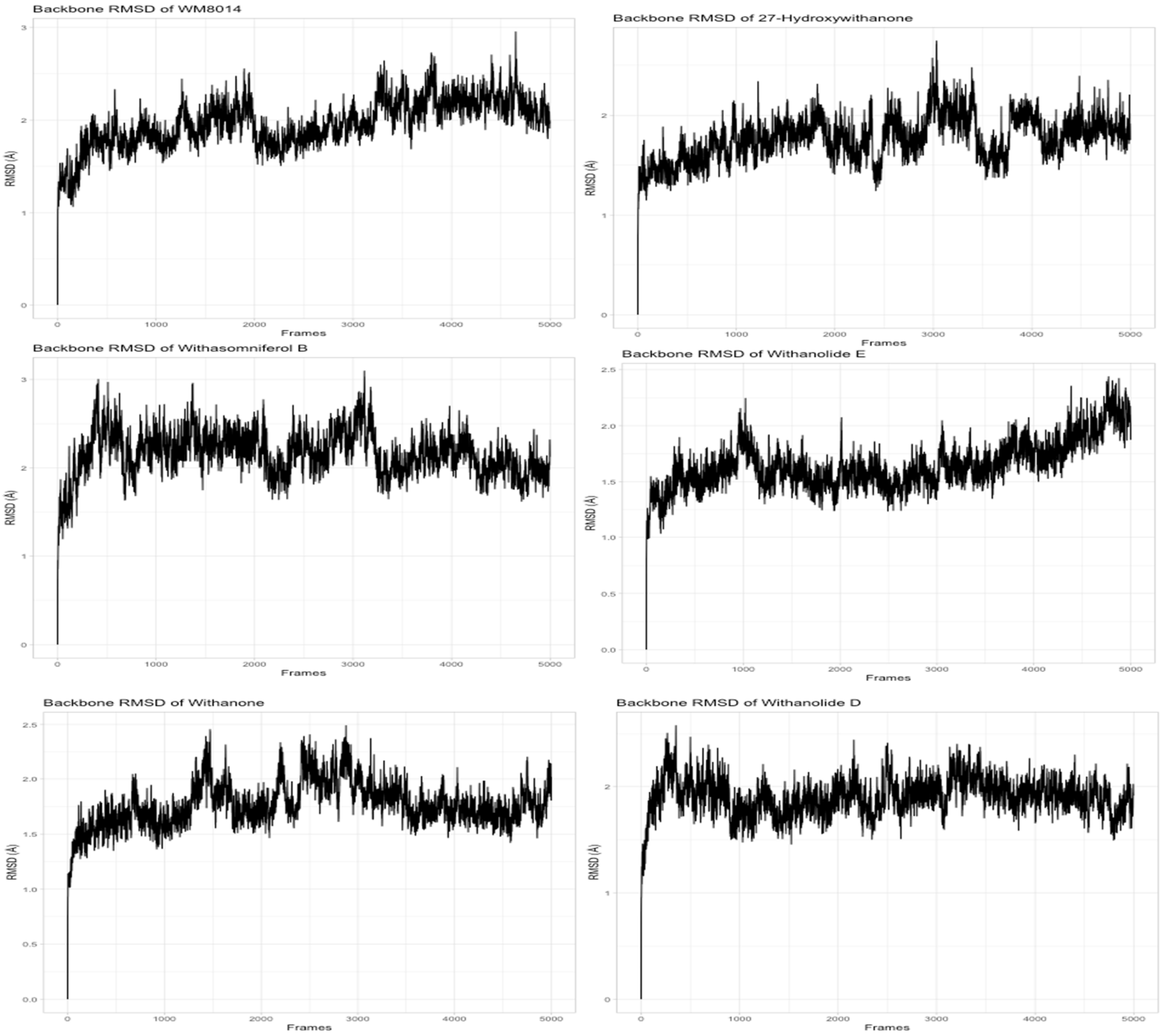
Backbone RMSD of WM8014
Backbone RMSD of 27-Hydroxywithanone
Backbone RMSD of Withasomniferol B
Backbone RMSD of Withanolide E
Backbone RMSD of Withanone
Backbone RMSD of Withanolide D
RMSD (Å)
Frames

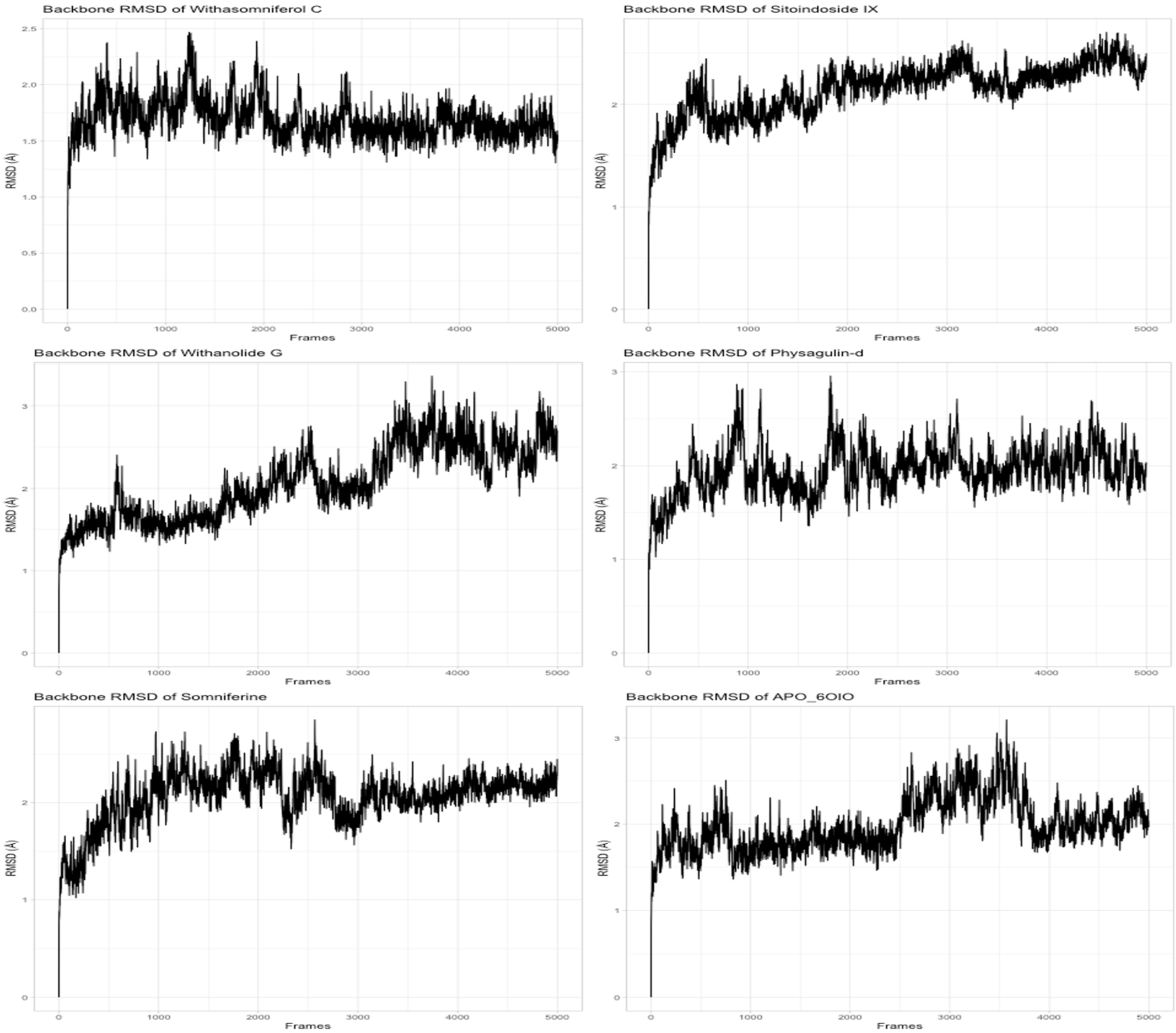

Supplementary Figure S2: Individual RMSD plots of protein backbone from Molecular dynamics for all the protein-ligand complexes

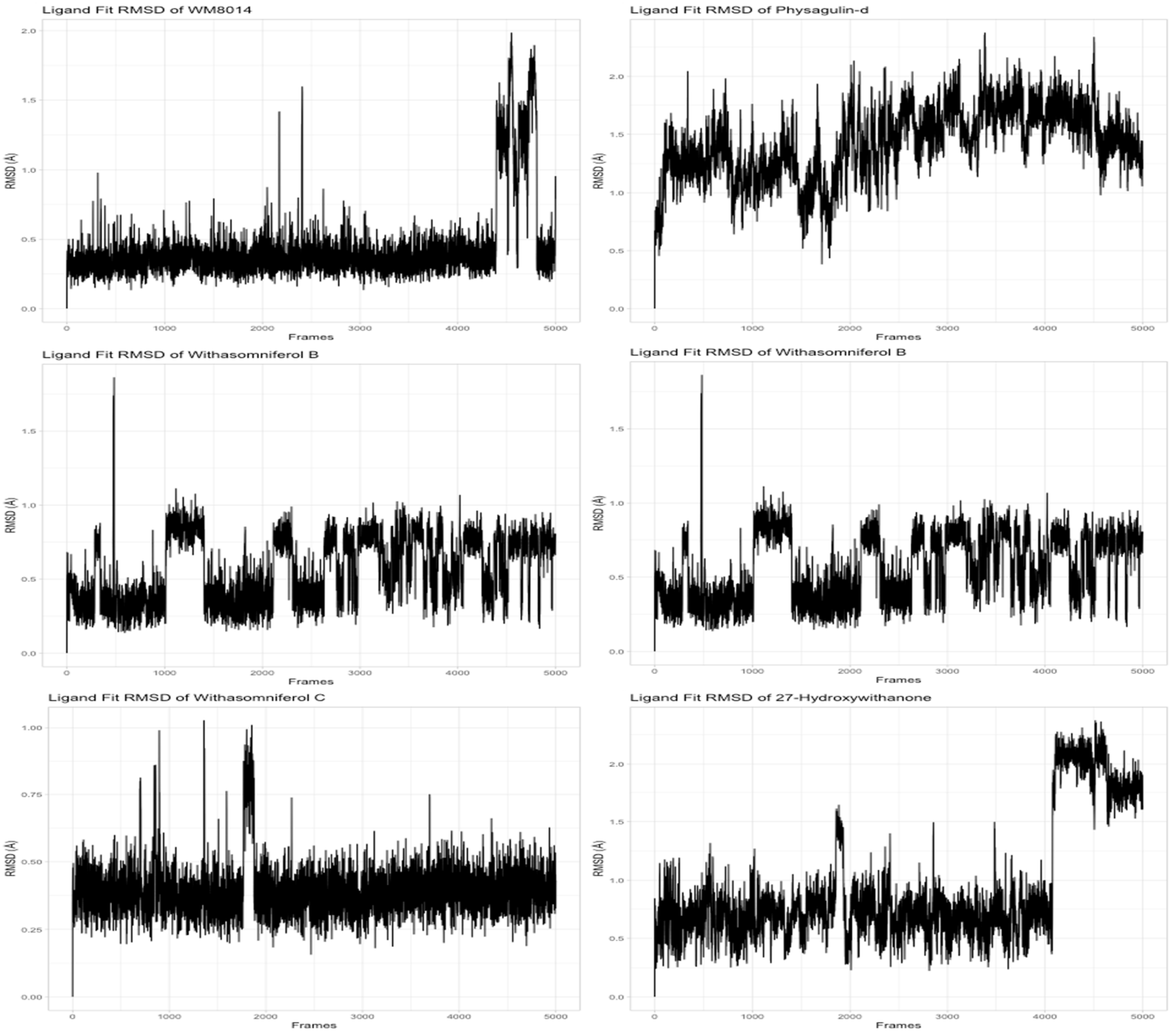
Ligand Fit RMSD of WM8014
Ligand Fit RMSD of Physagulin-d
Ligand Fit RMSD of Withasomniferol B
Ligand Fit RMSD of Withasomniferol B
Ligand Fit RMSD of Withasomniferol C
Ligand Fit RMSD of 27-Hydroxywithanone
RMSD (A)
Frames
0
1000
2000
3000
4000
5000
0.0
0.5
1.0
1.5
2.0
0.00
0.25
0.50
0.75
1.00

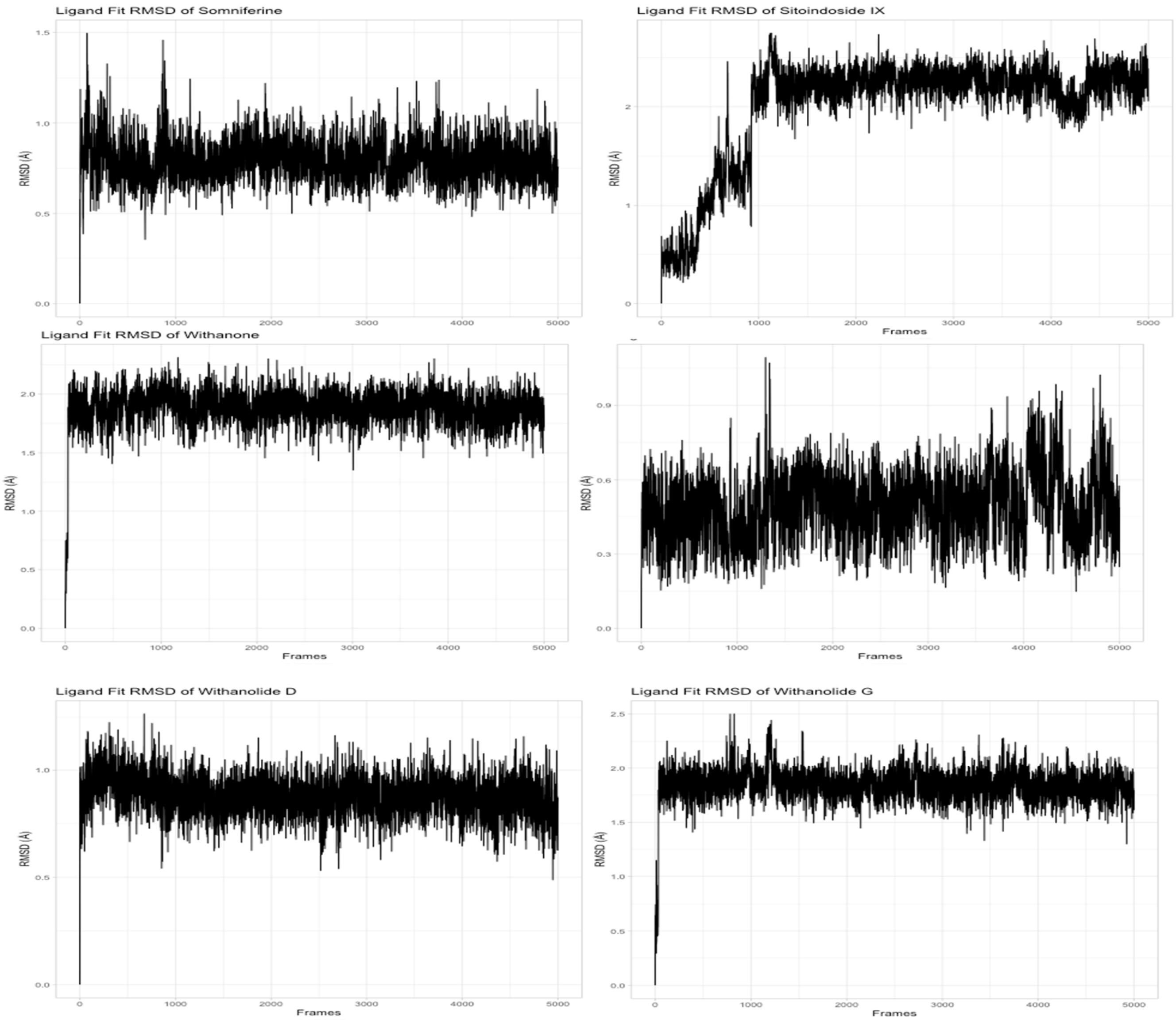

Supplementary Figure S3: Individual RMSD plot for ligands from Molecular dynamics for all the phytocompounds and the inhibitor

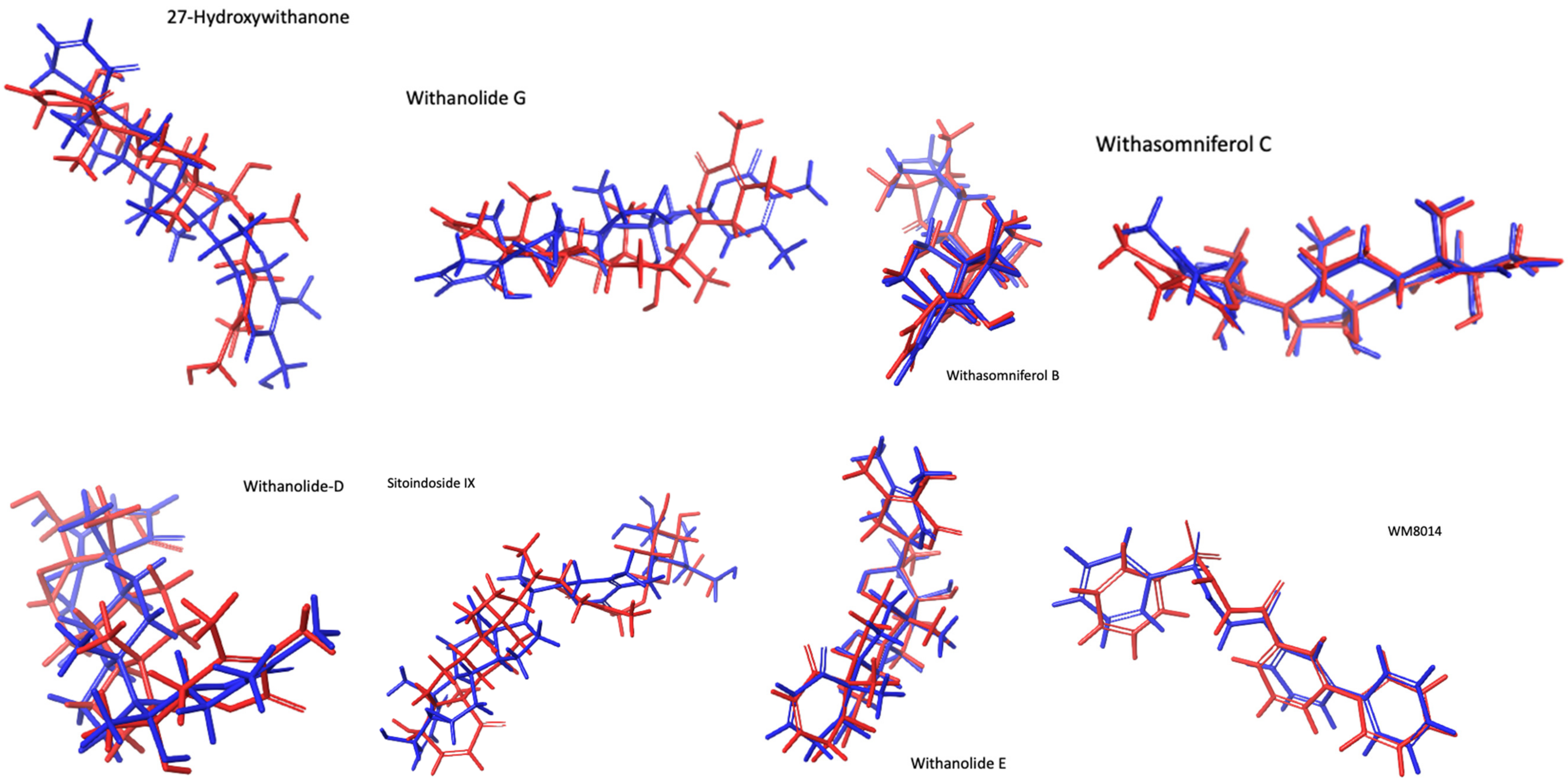

Supplementary Figure S4: Superimposed structures of compounds (First and last frame of molecular dynamics) from Withania somnifera which showed promising results with RMSF values.

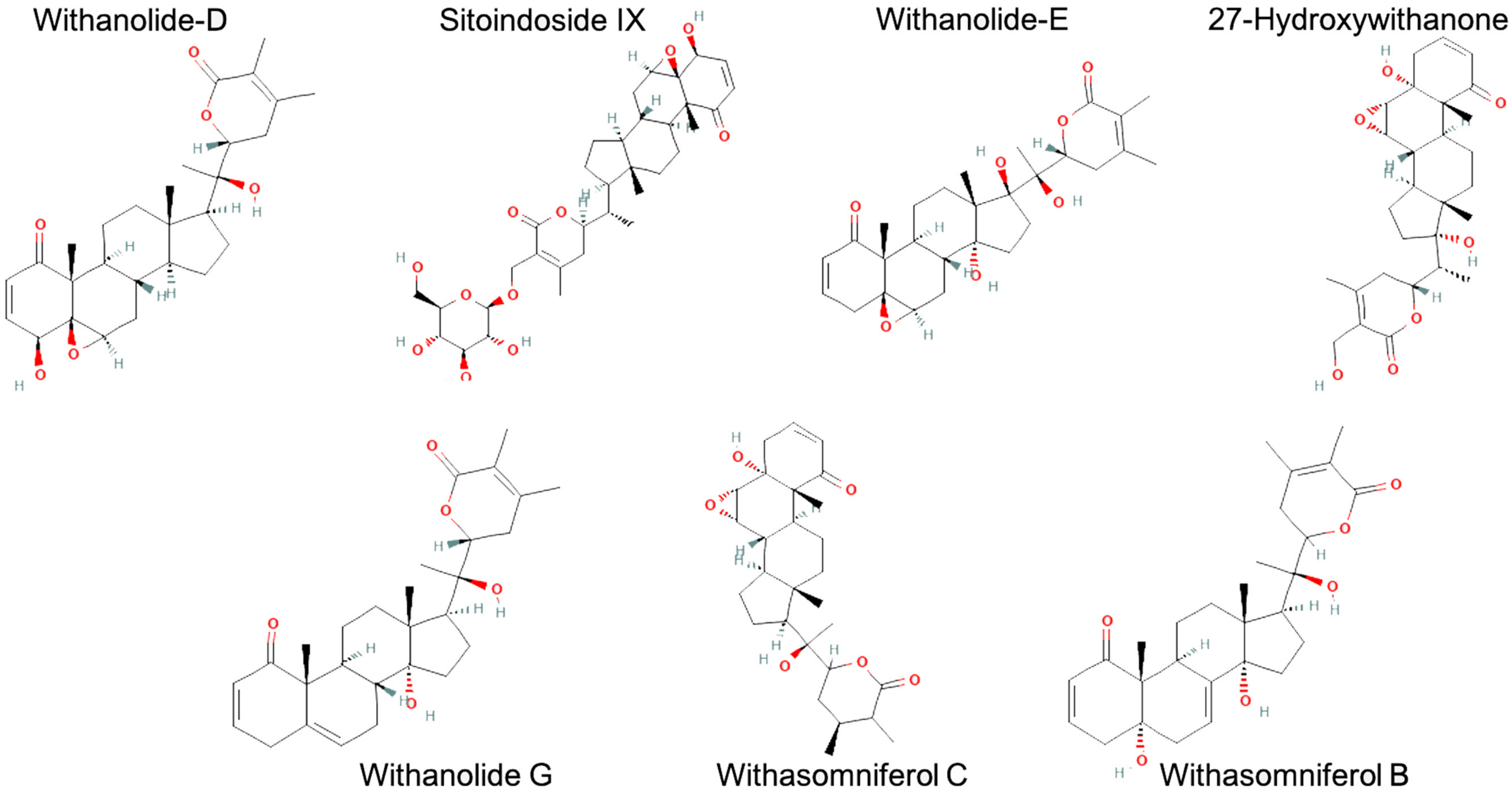

Supplementary Figure S5: 2D structures of compounds) from Withania somnifera subjected to molecular dynamics.

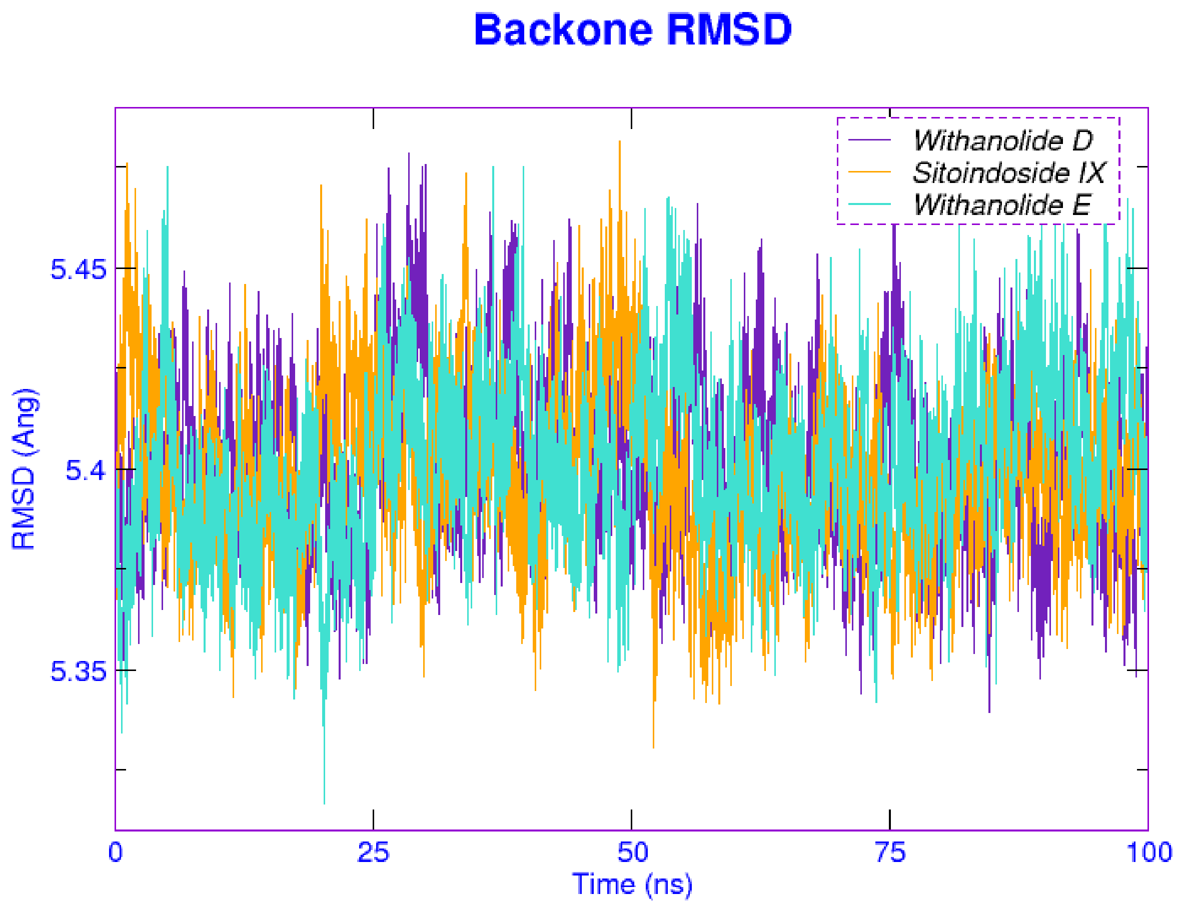

Supplementary Figure S6: RMSD plots of Withanolide D, Withanolide E and Sitoindoside IX in complex KAT6A systems for duration of 100 ns.

Supplementary TableS1: Predicted physicochemical properties of phytocompounds and standard drug.

**Name**	**Molecular weight**	**Log P**	**Surface Area**	**Water solubility**	**Caco2 permeability**	**Intestinal absorption (human)**	**VDss (human)**	**Fraction unbound (human)**	**BBB permeability**	**CNS permeability**	**Total Clearance**	**Oral Rat Acute Toxicity (LD50)**	**Hepatotoxicity**
Somniferine	608.691	2.6981	260.855	-3.12	1.53	94.052	1.633	0.262	-0.548	-3.073	1.051	3.111	No
Sitoindoside IX	632.747	1.1771	263.111	-4.419	0.412	55.154	-0.626	0.317	-1.183	-3.703	0.459	3.891	No
Withasomniferol B	472.622	3.4312	202.007	-4.674	0.87	100	-0.173	0.09	-0.254	-2.613	0.292	3.009	No
Withanolide G	454.607	4.4283	196.52	-5.463	0.977	95.969	0.221	0.131	-0.191	-2.894	0.434	2.108	No
Withanone	470.606	3.4954	201.317	-5.127	0.849	100	0.081	0.154	-0.259	-2.719	0.385	2.907	No
Withanolide E	486.605	2.7544	206.111	-5.252	0.742	100	0.093	0.286	-0.956	-3.098	0.378	3.503	No
Physagulin-d	620.78	1.9817	259.636	-3.959	0.429	54.898	-0.813	0.292	-1.066	-3.532	0.615	2.773	No
Withasomniferol C	470.606	3.5432	201.314	-5.269	0.769	97.917	0.142	0.23	-0.774	-3.008	0.43	2.334	No
Withanolide D	470.606	3.4954	201.317	-5.127	0.831	99.2	-0.048	0.093	-0.315	-2.696	0.347	2.831	No
27-Hydroxywithanone	486.605	2.4678	206.111	-4.865	0.759	88.231	-0.043	0.256	-0.548	-3.022	0.456	3.314	No
Withasomniferol A	486.605	2.4678	206.111	-4.81	0.756	88.275	-0.249	0.195	-0.505	-2.983	0.408	3.278	Yes
Withanolide A	470.606	3.4954	201.317	-5.096	0.846	100	-0.097	0.096	-0.255	-2.681	0.337	2.913	No
Withanoside VI	782.921	-0.0516	321.429	-2.974	-0.684	39.364	-0.563	0.408	-1.595	-4.412	0.544	2.875	No
Withanoside V	766.922	0.8335	316.635	-3.056	-0.579	45.414	-0.601	0.364	-1.524	-4.134	0.563	2.912	No
Withaferin A	470.606	3.3529	201.317	-5.063	0.829	85.345	-0.131	0.105	-0.03	-2.72	0.435	2.779	No
Withanoside IV	782.921	-0.1941	321.429	-2.887	-0.631	31.451	-0.605	0.398	-1.51	-4.436	0.636	2.886	No
Sitogluside	576.859	5.849	248.833	-4.741	0.472	79.677	-1.163	0.078	-0.785	-3.021	0.689	2.571	No
(-)-Anaferine	224.348	1.6199	98.368	-1.204	1.336	93.738	0.908	0.754	0.112	-3.249	1.272	2.483	No

Withasomnine	184.242	2.4963	83.185	-2.402	1.422	97.188	0.272	0.277	0.289	-1.699	0.34	2.093	No
Cuscohygrine	224.348	1.5241	98.788	-1.108	1.364	94.096	0.979	0.757	0.236	-3.226	1.159	2.385	No
Anahygrine	224.348	1.572	98.578	-1.121	1.349	93.917	0.957	0.755	0.225	-3.237	1.218	2.407	No
Nicotine	162.236	1.8483	73.23	-0.872	1.671	95.867	0.595	0.628	0.208	-2.995	0.86	2.432	Yes
Pelletierine	141.214	1.1076	61.999	-0.426	1.363	97.349	0.431	0.774	0.063	-2.997	1.073	2.249	Yes
Hygrine	141.214	1.0597	62.209	-0.439	1.378	100	0.453	0.777	0.107	-2.985	1.002	2.224	Yes
Tropine	141.214	0.6039	61.836	-0.514	1.444	93.575	0.54	0.796	-0.023	-3.339	1.063	1.91	Yes
Galactitol	182.172	-3.5854	69.329	-1.151	-0.441	25.401	-0.325	0.868	-1.309	-4.13	0.919	1.637	No
WM8014	384.432	3.42442	156.433	-5.355	1.185	92.249	-1.044	0	0.066	-2.304	0.451	2.017	Yes

Supplementary Table S2: RMSF values of compounds before and after simulation having the best results.

**Compound Name**	**RMSF**
Withanolide-D	0.8715
Sitoindoside IX	2.0561
WITHANOLIDE E	0.4904
27-Hydroxywithanone	1.6391
Withanolide G	1.7874
Withasomniferol C	0.4262
Withasomniferol B	0.8036
WM8014	1.5051
